# Factors Associated with Postoperative Hemorrhage in Primary Metabolic Bariatric Surgery: Nationwide Analysis of Patient Characteristics, Surgical and Stapling Techniques

**DOI:** 10.1007/s11695-026-08930-9

**Published:** 2026-10-02

**Authors:** Anne J. Brouwer, Mabel M.A. Kwakkelstein, Jan A. Apers, Floris F.E. Bruinsma, Martin Dunkelgrün, Bo J. Noordman, Guusje Vugts, Simon W. Nienhuijs, Ronald S.L. Liem, Dutch Audit for Treatment of Obesity Research Group

**Affiliations:** 1 Dutch Institute for Clinical Auditing, Leiden, Netherlands; 2 Department of Surgery, NUTRIM Institute of Nutrition and Translational Research in Metabolism, Maastricht University, Maastricht, Netherlands; 3 Department of Surgery, Franciscus Gasthuis & Vlietland, Rotterdam, Netherlands; 4 Department of Surgery, Amsterdam University Medical Centers, Amsterdam, Netherlands; 5 Department of Surgery, Ziekenhuis Gelderse Vallei, Ede, Netherlands; 6 Department of Surgery, Catharina Ziekenhuis, Eindhoven, Netherlands; 7 Department of Surgery, Groene Hart Ziekenhuis, Gouda, Netherlands; 8 Nederlandse Obesitas Kliniek, Den Haag en Gouda, Netherlands

**Keywords:** Metabolic Bariatric Surgery, Postoperative Hemorrhage, Surgical techniques, Tranexamic Acid, Thromboprophylaxis regimens

## Abstract

**Introduction:**

Postoperative hemorrhage is the most common severe complication following metabolic bariatric surgery (MBS). Evidence on associations with surgical and stapling techniques, tranexamic acid (TXA) use, and thromboprophylaxis regimens remains scarce. The aim of this study is to evaluate patient characteristics, surgical and stapling techniques, thromboprophylaxis regimens, and TXA use as factors associated with postoperative hemorrhage following primary MBS.

**Methods:**

Patients undergoing primary MBS between 2015 and 2024 were selected from the Dutch Audit for Treatment of Obesity (DATO). Surgical details, including surgical and stapling techniques, stapler brand, thromboprophylaxis regimens, and TXA protocols, were retrospectively collected via nationwide questionnaires. The primary outcome was postoperative hemorrhage (Clavien Dindo (CD) grade ≥ 2) within 30 days of the procedure. Secondary outcomes included reinterventions, intensive care unit admission, mortality, and length of hospital stay. Multilevel logistic regression analysis was performed to identify factors associated with postoperative hemorrhage of CD grade ≥ 2.

**Results:**

A total of 83,580 patients were included, with 18,701 sleeve gastrectomies (SG) and 64,879 bypass procedures. Postoperative hemorrhage of CD grade ≥ 2 occurred in 1.2% (n = 1021). The following factors were independently associated with postoperative hemorrhage: BMI (kg/m2) (adjusted odds ratio [aOR] = 0.974; 95% confidence interval [CI]: 0.957–0.991), stroke or transient ischemic attack in medical history (aOR = 1.751; 95% CI: 1.097–2.796), hypertension (aOR = 1.341; 95% CI: 1.080–1.665), and liver disease (aOR = 1.838; 95% CI: 1.038–3.253).

**Conclusion:**

Despite limitations inherent in its retrospective, questionnaire-based design, the findings indicate that patient-related factors are associated with postoperative hemorrhage risk in MBS.

**Key points:**

• Postoperative hemorrhage is the most common severe short term complication following MBS.

• Most postoperative hemorrhages require surgical reintervention.

• There is no association between surgical techniques and postoperative hemorrhage.

**Supplementary Information:**

The online version contains supplementary material available at https://doi.org/10.1007/s11695-026-08930-9.

## Introduction

In recent years, the number of metabolic and bariatric procedures performed worldwide has continued to increase [1, 2]. This rising demand for metabolic and bariatric surgery (MBS) has resulted in a substantial growth of fast-track pathways and day-care surgery.

Over the past few decades, the safety of MBS has markedly improved due to the introduction of laparoscopy, centralization of care, and standardized perioperative protocols, including Enhanced Recovery After Bariatric Surgery (ERABS) protocols.

Despite these improvements, complications still occur. Postoperative hemorrhage is the most common early complication, occurring in 0.8–3.8% of patients [3–6] and is associated with prolonged hospitalization, ICU admission, reintervention, and increased mortality [4]. In most cases, bleeding originates from the staple lines, but it may also originate from ligated vessels, the omentum, dissected planes, or the abdominal wall. Postoperative hemorrhage is often self-limiting. However, in some cases it may require urgent surgical, radiological, or endoscopic treatment [3].

While fast-track and day-care surgery offer many benefits, the shorter diagnostic windows may inadvertently increase the risk of adverse outcomes from postoperative hemorrhage. Therefore, early recognition and understanding of hemorrhage risk factors are crucial [7]. This could reduce reintervention rates and healthcare costs while enhancing patient recovery and overall surgical outcomes.

Recent studies have identified male sex, older age, hypertension, renal insufficiency, thromboembolic disease, and perioperative anticoagulant use as predictors of postoperative hemorrhage [4, 7]. Although studies have compared complication rates between powered and manual stapling devices in gastrointestinal and thoracic surgery [8], literature focused on stapling devices and techniques in MBS is scarce. In addition, the role of tranexamic acid (TXA) has been studied for sleeve gastrectomy (SG) [9, 10], but evidence regarding its use in bypass procedures remains limited. Moreover, there are no studies reporting on the association between postoperative hemorrhage and specific surgical practices, such as variation in stapling materials and techniques, use of TXA, or thromboprophylaxis regimens employed in MBS. Therefore, this study aims to identify risk factors associated with postoperative hemorrhage in patients undergoing primary MBS by evaluating the association with patient characteristics, comorbidities, surgical technique, stapler material, thromboprophylaxis regimens, and TXA use.

## Methods

This study was performed using data from the Dutch Audit for Treatment of Obesity (DATO), a prospective Dutch National Registry of pseudo-anonymized data that includes demographics, treatment characteristics, and follow-up data for all patients undergoing MBS in the Netherlands. The study protocol was reviewed and approved by a scientific committee from Dutch Institute for Clinical Auditing (DICA), and this specific registry, which consists of mandated surgeons from all Dutch hospitals where MBS is performed[11] [12].

### Patients – Selection and Inclusion

All primary SG procedures and Gastric Bypass procedures (including Roux-en-Y Gastric Bypass [RYGB], One-Anastomosis Gastric Bypass [OAGB], and ring-augmented RYGB [RA-RYGB] and OAGB [RA-OAGB]) procedures between January 2015 and December 2024 were eligible for inclusion.

### Data Acquisition

Baseline characteristics, surgical specifications, and clinical outcomes were derived from the DATO database. Demographic data included age, sex, preoperative weight, BMI and comorbidities.

Additional surgical specifications were collected retrospectively via detailed questionnaires sent to all bariatric centers in the Netherlands. These included surgical techniques, whether omentopexy was performed in SG, type and brand of stapling devices, perioperative TXA protocols, and perioperative thromboprophylaxis regimens, including duration, agent, and dosage. The stapling material, collected by the questionnaire, was verified using order histories provided by the various stapling device manufacturers.

Whenever adjustments were made in each of these treatment protocols during the timeframe of this study, detailed information was gathered on the timeline of these changes. Using the timeline and filled-out questionnaires, each procedure in DATO was matched to the specific surgical techniques, thromboprophylaxis and TXA protocols at that time based on the treatment center and the operation date in DATO.

### Endpoints

The primary endpoint was the postoperative hemorrhage rate (Clavien Dindo [CD] ≥ 2) within 30 days of the procedure. Secondary endpoints were surgical, endoscopic, or radiological reinterventions, intensive care admissions, readmissions, mortality, and length of hospital stay.

### Statistical Analysis

Data distributions were assessed using histograms. Each outcome was categorized per bariatric technique, and expressed in proportions, means, and standard deviation (SD), or medians and ranges where appropriate. In case of missing data, it was assessed whether data were missing at random or missing not at random. If necessary and possible, missing data were imputed.

A univariable analysis was performed to evaluate factors associated with a CD grade ≥ 2 postoperative hemorrhage. Subsequently, a multivariable analysis was conducted to assess independent associations of these variables. Variables for the multivariable model were selected based on the univariable tests (p-value ≤ 0.1), data quality, and clinical relevance. Data quality was the determining factor in excluding the duration of thromboprophylaxis and preoperative thromboprophylaxis from the final analysis.

Multiple centers expressed uncertainty about the timeframe during which certain thromboprophylaxis protocols were introduced or altered. In addition, the thromboprophylaxis protocols could not be verified. Upon further inquiry, all centers were able to confirm that the thromboprophylaxis agent did not change during the course of this study, and this variable was therefore included in the final analysis. However, to assess the robustness of our findings, a sensitivity analysis was performed, excluding all variables related to thromboprophylaxis (Supplementary material 1).

Multilevel analysis was performed to examine the data for clustering. Based on the intraclass correlation coefficient (ICC) and the difference in variance at the individual and hospital-level, the final decision between a multivariable and multilevel analysis was made. In the multivariable or multilevel analysis, a p-value < 0.05 was considered statistically significant. Statistical analyses were conducted using R version 4.4.0.

## Results

### Baseline Characteristics

**Fig. 1:**
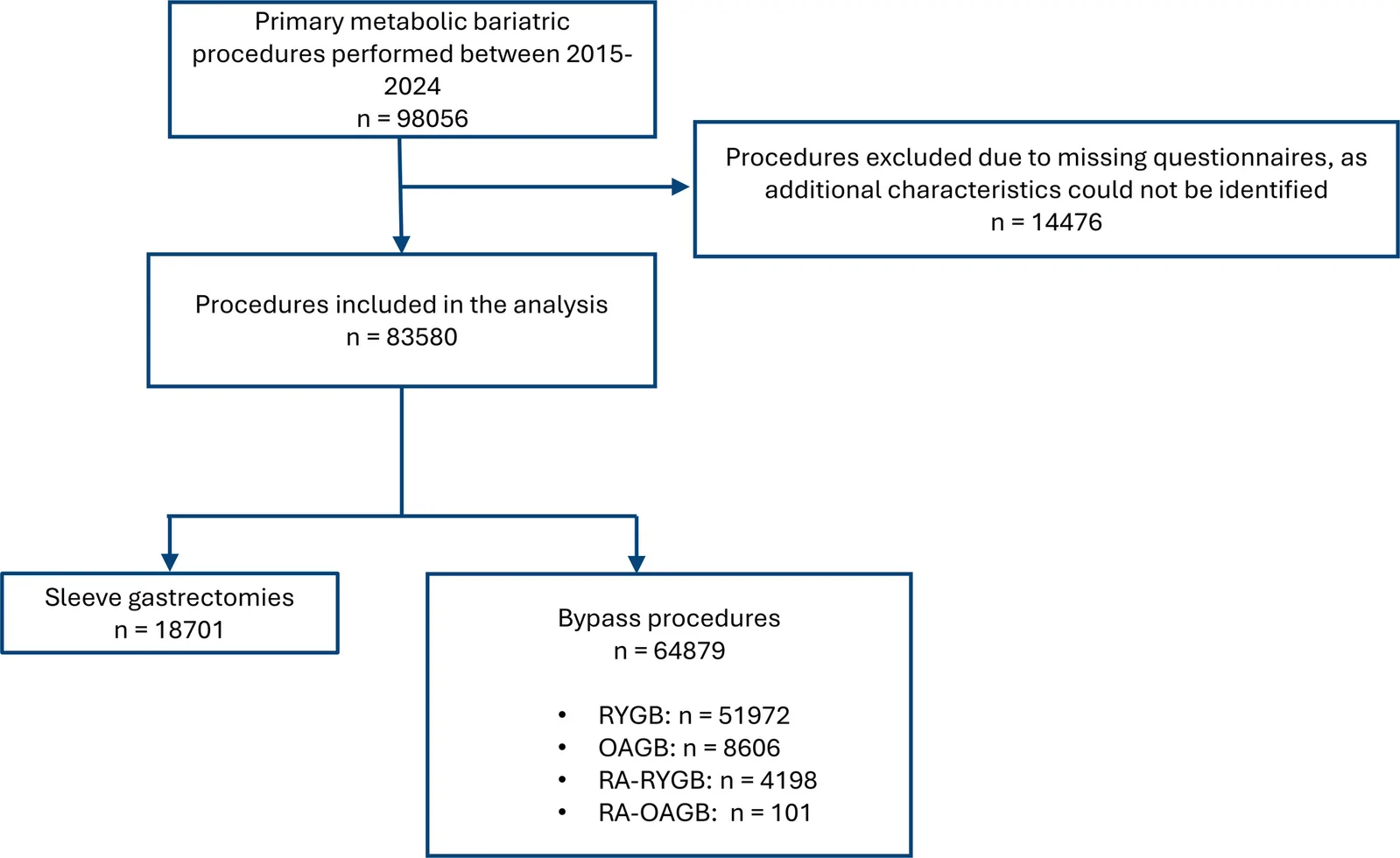
Flowchart. Abbreviations: RYGB = Roux-en-Y Gastric Bypass; OAGB = One-Anastomosis Gastric Bypass; RA-RYGB = ring-augmented Roux-en-Y Gastric Bypass; RA-OAGB = ring-augmented One-Anastomosis Gastric Bypass

Between 2015 and 2024, 98,056 primary metabolic and bariatric procedures were performed. 14,476 were excluded due to missing questionnaires. Therefore, 83,580 procedures were included in this study, comprising 18,701 (22.4%) SG and 64,879 bypass procedures (77.6%) (Fig. 1). Baseline characteristics are presented in Table 1.

**Table 1:** Baseline Characteristics

	Total (N = 83,580)	Sleeve gastrectomy (N = 18,701)	Bypass variations* (N = 64,879)	p-value
Sex (female)	65,842 (78.8)	13,996 (74.8)	51,846 (79.9)	< 0.001
Age, median ± IQR	45.0 (35.0–53.0)	42.0 (31.0–52.0)	46.0 (36.0–53.0)	< 0.001
BMI (kg/m2), median ± IQR	41.7 (39.3–45.3)	42.5 (40.0–46.7)	41.5 (39.1–45.0)	< 0.001
ASA score				< 0.001
I	836 (1.1)	191 (1.0)	705 (1.1)	
II	27,243 (32.6)	4779 (25.6)	22,464 (34.6)	
III	54,509 (65.2)	13,387 (71.6)	41,122(63.4)	
IV	623 (0.7)	276 (1.5)	347 (0.5)	
Diabetes	14,436 (17.3)	2497 (13.4)	11,939 (18.4)	< 0.001
Hypertension	27,298 (32.7)	5556 (29.7)	21,742 (33.5)	< 0.001
OSAS	15,068 (18.0)	3314 (17.7)	11,754 (18.1)	0.087
Dyslipidaemia	16,104 (19.3)	3252 (17.4)	12,852 (19.8)	< 0.001
Myocardial infarction in medical history	1342 (1.6)	275 (1.5)	1067 (1.6)	< 0.001
Congestive heart failure	1302 (1.6)	236 (1.3)	1066 (1.6)	< 0.001
Peripheral arterial disease/AAA	437 (0.5)	121 (0.6)	316 (0.5)	< 0.001
Stroke/TIA in medical history	1171 (1.4)	255 (1.4)	916 (1.4)	0.001
Increased risk VTE/PE**	319 (0.4)	87 (0.5)	232 (0.4)	0.005
Liver disease	532 (0.6)	154 (0.8)	378 (0.6)	< 0.001
Kidney disease	307 (0.4)	130 (0.7)	177 (0.3)	< 0.001
Malignancy in medical history	665 (0.8)	148 (0.8)	517 (0.8)	0.004
Paraplegia or hemiplegia	44 (0.1)	7 (0.0)	37 (0.1)	0.003

Data are expressed as number (%) of participants unless otherwise indicated. Abbreviations: IQR = inter quartile range; BMI = body mass index; kg/m^2^ = kilogram per squared meters; OSAS = obstructive sleep apnoea syndrome; ASA = American Society of Anesthesiologists; AAA = abdominal aortic aneurysm; TIA = transient ischemic attack; VTE = venous thromboembolism; PE = pulmonary embolism. *Bypass variations: Roux-en-Y gastric bypass, one anastomosis gastric bypass and ring-augmented RYGB and OAGB. ** Increased risk of VTE/PE was defined as a venous thromboembolism or pulmonary hypertension in medical history, hypoventilation due to obesity, or when a peroperative vena cava filter was used

### Surgical Outcomes

Surgical outcomes are presented in Table 2. There were 1.273 postoperative hemorrhages recorded (CD grades 1–5), accounting for a postoperative hemorrhage rate of 1.5%. Postoperative hemorrhage rate with CD grade ≥ 2 was 1.2% (n = 1.021). Surgical reintervention was indicated for 232/348 postoperative hemorrhages (67%) following SG and for 286/925 (30.9%) following bypass procedures. Postoperative deep vein thrombosis or pulmonary embolism was observed in 45 cases (0.05%), of which 15 occurred following SG (0.08%) and 30 following bypass procedures (0.05%). Two of the postoperative hemorrhage cases (0.6%) following SG were fatal, compared to 5 (0.5%) following bypass procedures.

**Table 2:** Surgical outcomes

	Total (N = 83,580)	Sleeve gastrectomy (N = 18,701)	Bypass variations* (N = 64,879)	p-value
Length of stay (in days), median ± IQR	1.0 (1.0–1.0)	1 (1.0–1.0)	1 (1.0–1.0)	< 0.001c
Length of stay (in days) in patients with postoperative hemorrhage, median ± IQR	3.0 (1.0–5.0)	4.0 (3.0–5.0)	3.0 (1.0–4.0)	< 0.001c
Postoperative deep vein thrombosis or pulmonary embolism	45 (0.05)	15 (0.08)	30 (0.05)	0.111a
Postoperative hemorrhage rate				
Total	1273 (1.5)	348 (1.9)	925 (1.4)	< 0.001a
CD 1	126/1147 (11.0)	17/330 (5.2)	109/817(13.3)	
CD 2	402/1147 (35.0)	71/330 (21.5)	331/817 (40.5)	
CD 3	526/1147 (45.9)	217/330 (65.8)	309/817 (37.8)	
CD 4	89/1147 (7.8)	24/330 (7.3)	65/817 (8.0)	
CD 5	4/1147 (0.3)	1/330 (0.3)	3/817 (0.4)	
CD Missing	126	18	108	
Reintervention rate due to bleeding complication				
Total	621/1147 (54.1)	240/348 (69.0)	381/925 (41.2)	< 0.001a
Surgical procedure	518/621 (83.4)	232/240 (96.7)	286/381 (75.1)	
Endoscopic procedure	79/621 (12.7)	5/240 (2.1)	74/381 (19.4)	
Interventional radiology procedure	5/621 (0.8)	1/240 (0.4)	4/381 (1.0)	
Other	19/621 (7.3)	2/240 (0.8)	17/381 (4.5)	
ICU admission due to bleeding complication	49/1147 (4.3)	16/348 (4.6)	33/925 (3.6)	0.836a
Readmission rate due to bleeding complication	244/1147 (21.3)	34/348 (9.8)	210/925 (22.7)	< 0.001a
Mortality due to bleeding complication	7/1147 (0.6)	2/348 (0.6)	5/925 (0.5)	0.264b

Data are expressed as number (%) of participants unless otherwise indicated. Abbreviations: IQR = inter quartile range; CD = Clavien-Dindo classification; ICU = intensive care unit. *Bypass variations: Roux-en-Y gastric bypass, one anastomosis gastric bypass and ring-augmented RYGB and OAGB. ^a^ Chi-Squared between groups. ^b^ Fisher exact. ^c^ Mann–Whitney U-test

The questionnaire results are presented in Table 3. Powered staplers were used for the majority of procedures (n = 49,801, 59.6%). In 2.5% of SG procedures, TXA was administered routinely at the start of the procedure. The duration of thromboprophylaxis ranged from only during hospital admission to > 4 weeks after surgery. Prolonged thromboprophylaxis (> 4 weeks) was predominantly used in procedures that were performed between 2015 and 2020 (n = 24,075, 60.7%). The majority of patients received a double dose of thromboprophylaxis (i.e., 5.000 IE dalteparin or 5.700 IE nadroparin) (n = 76,749, 91.8%).

**Table 3:** Questionnaire outcomes—surgical techniques and thromboprophylaxis regimens

	Total (N = 83,580)	Sleeve gastrectomy (N = 18,701)	Bypass variations* (N = 64,879)	p-value
Brand Stapler				< 0.001a
A	57,437 (68.7)	10,830 (57.9)	46,607 (71.8)	
B	23,931 (28.6)	7335 (39.2)	16,596 (25.6)	
C	2212 (2.6)	536 (2.9)	1676 (2.6)	
Type of stapling device				< 0.001a
Powered	49,801 (59.6)	11,436 (61.2)	38,365 (59.1)	
Manual	33,275 (39.8)	7196 (38.5)	26,079 (40.2)	
Standardized TXA pre- or peroperatively	475 (0.6)	475 (2.5)	0 (0.0)	< 0.001a
Omentopexy in Sleeve Gastrectomy	452 (0.5)	452 (2.4)	NA	UTD
Preoperative thromboprophylaxis	17,775 (21.3)	3467 (18.5)	14,308 (22.1)	< 0.001a
Duration thromboprophylaxis				< 0.001a
Only during hospital admission	21,760 (26.0)	5321 (28.5)	16,439 (25.3)	
1 week	8757 (10.5)	1049 (5.6)	7708 (11.9)	
2 weeks	8474 (10.1)	1502 (8.0)	6972 (10.7)	
3 weeks	4949 (5.9)	2050 (11.0)	2899 (4.5)	
4 or more weeks	39,639 (47.4)	8779 (46.9)	30,860 (47.6)	
Tromboprophylaxis agent				< 0.001a
Dalteparin	37,696 (45.1)	9577 (51.2)	28,119 (43.3)	
Nadroparin	36,033 (43.1)	6441 (34.4)	29,592 (45.6)	
Dosage thromboprophylaxis				< 0.001a
Single dose	261 (0.3)	19 (0.1)	242 (0.4)	
Double dose	76,749 (91.8)	15,067 (80.6)	61,682 (95.1)	
Therapeutic dose	28 (0.0)	8 (0.0)	20 (0.0)	
7500EH dalteparin	6542 (7.8)	3607 (19.3)	2935 (4.5)	

Data are expressed as number (%) of participants unless otherwise indicated. Abbreviations: TXA = tranexamic acid. *Bypass variations: Roux-en-Y gastric bypass, one anastomosis gastric bypass and ring-augmented RYGB and OAGB. ^a^ Chi-Squared between groups. ^b^ Fisher exact

### Univariate and Multilevel Logistic Regression Analysis

Outcomes of the univariate analysis are presented in Table 4. A multilevel analysis was performed to assess whether clustering of data at the hospital level was present. The intraclass correlation coefficient (ICC) in the random-intercept null model was 0.045, indicating modest clustering at the hospital level. After adjustment for all covariates included in the multilevel analysis, the between-hospital variance decreased from 0.155 to 0.098 (ICC = 0.029), suggesting that part of the between-hospital variation was explained by the included variables. Therefore, multilevel logistic regression analysis was performed. The multilevel analysis was based on an event rate of 1.021 postoperative hemorrhages (CD grade ≥ 2). Multilevel analysis showed the following significant associations with postoperative hemorrhage in MBS: BMI (kg/m2) (adjusted odds ratio [aOR] = 0.974; 95% confidence interval [CI]: 0.957–0.991), stroke or transient ischemic attack (TIA) in medical history (aOR = 1.751; 95% CI: 1.097–2.796), hypertension (aOR = 1.341; 95% CI: 1.080–1.665), and liver disease (aOR = 1.838; 95% CI: 1.038–3.253).

**Table 4:** Univariable and multilevel analysis

Predictors		Crude odds ratio	95% CI OR	p-value	Adjusted odds ratio	95% CI OR	p-value
Sex	Male (ref)	Ref			Ref		
	Female	0.633	0.554—0.726	< 0.001	0.876	0.699—1.098	0.251
Age (per year)		1.024	1.018- 1.029	< 0.001	1.009	1.000—1.020	0.070
BMI (kg/m2) (for each BMI point)		0.965	0.953–0.978	< 0.001	0.974	0.957—0.991	0.010
Type of surgery	Sleeve gastectomy	Ref			Ref		
	Bypass variation*	0.647	0.567–0.741	< 0.001	0.651	0.520—0.816	0.074
ASA score	1	Ref					
	2	1.443	0.736–3.388	0.339			
	3	1.643	0.842–3.843	0.193			
	4	1.862	0.690–5.234	0.220			
Increased risk VTE/PE**	No increased risk	Ref					
	Increased risk	1.158	0.410–2.538	0.748			
Stroke/TIA in medical history	No	Ref			Ref		
	Yes	1.973	1.313–2.842	< 0.001	1.751	1.097—2.796	0.019
Peripheral arterial disease/AAA	No	Ref			Ref		
	Yes	2.639	1.468–4.350	< 0.001	1.601	0.858—2.987	0.509
Diabetes	No	Ref			Ref		
	Yes	1.338	1.149–1.551	< 0.001	1.158	0.902—1.487	0.251
Hypertension	No	Ref			Ref		
	Yes	1.605	1.417–1.818	< 0.001	1.341	1.080—1.665	0.008
Kidney disease	No	Ref					
	Yes	1.307	0.465–2.850	0.555			
Myocardial infarction in medical history	No	Ref			Ref		
	Yes	2.192	1.531–3.039	< 0.001	1.273	0.777—2.086	0.337
Liver disease	No	Ref			Ref		
	Yes	2.157	1.201–3.550	0.005	1.838	1.038—3.253	0.037
Malignancy in medical history	No	Ref					
	Yes	1.333	0.686–2.314	0.349			
OSAS	No	Ref			Ref		
	Yes	1.407	1.214–1.625	< 0.001	1.242	0.994—1.551	0.057
Dyslipidaemia	No	Ref			Ref		
	Yes	1.373	1.188–1.583	< 0.001	0.866	0.674—1.114	0.262
Congestive heart failure	No	Ref			Ref		
	Yes	1.724	1.148–2.481	0.005	1.047	0.620—1.768	0.863
Preoperative thromboprophylaxis	No	Ref					
	Yes	0.733	0.619–0.862	< 0.001			
Tromboprophylaxis agent	Dalteparin	Ref			Ref		
	Nadroparin	0.882	0.772–1.007	0.064	0.831	0.560—1.235	0.361
Duration thromboprophylaxis	Only during hospital admission	Ref					
	1 week	0.487	0.372–0.628	< 0.001			
	2 weeks	0.631	0.494–0.797	< 0.001			
	3 weeks	1.140	0.896–1.435	0.275			
	4 or more weeks	0.689	0.598–0.795	< 0.001			
Dosage thromboprophylaxis	Single dose	Ref					
	Double dose	1.510	0.484–9.123	0.562			
	Therapeutic dose	4.796	0.219–51.671	0.207			
	7500EH dalteparin	2.318	0.731–14.089	0.240			
Standardized TXA pre- or peroperatively	No standard use	Ref					
	Standard use	1.039	0.411–2.126	0.925			
Omentopexy in Sleeve Gastrectomy	No	Ref					
	Yes	0.905	0.323–1.963	0.824			
Brand stapling device	A	Ref			Ref		
	B	0.880	0.763–1.011	0.075	0.746	0.452—1.233	0.253
	C	0.780	0.494–1.164	0.254	0.968	0.550—1.701	0.909
Type of stapling device	Powered	Ref			Ref		
	Manual	0.873	0.767–0.991	0.037	0.888	0.582—1.355	0.581
Year	2015	Ref			Ref		
	2016	0.994	0.737–1.345	0.971	2.584	0.278—24.002	0.404
	2017	1.041	0.776–1.400	0.791	1.045	0.092—11.845	0.972
	2018	1.136	0.849–1.526	0.393	0.773	0.102—5.828	0.802
	2019	0.985	0.735–1.324	0.918	0.417	0.057—3.071	0.390
	2020	1.109	0.809–1.520	0.519	0.554	0.075—4.088	0.563
	2021	1.146	0.852–1.547	0.369	0.525	0.071—3.871	0.527
	2022	1.192	0.902–1.584	0.221	0.562	0.076—4.134	0.571
	2023	1.329	1.003–1.770	0.050	0.578	0.079—4.261	0.591
	2024	1.340	1.007–1.793	0.046	0.567	0.077—4.191	0.578

Univariable analyses present the unadjusted association between each predictor and the post-operative hemorrhage rate (CD ≥ 2). Variables for the multilevel were selected based on the results of the univariable tests (p-value ≤ 0.1), data quality and clinical relevance. Multilevel analysis included all relevant covariates simultaneously, yielding adjusted odds ratios that account for potential confounding

Abbreviations: CI = confidence interval; BMI = body mass index; kg/m^2^ = kilogram per squared meters; OSAS = obstructive sleep apnoea syndrome; ASA = American Society of Anesthesiologists; AAA = abdominal aortic aneurysm; TIA = transient ischemic attack; VTE = venous thromboembolism; PE = pulmonary embolism; TXA = tranexamic acid. *Bypass variations: Roux-en-Y gastric bypass, one anastomosis gastric bypass and ring-augmented RYGB and OAGB. ** Increased risk of VTE/PE was defined as a venous thromboembolism or pulmonary hypertension in medical history, hypoventilation due to obesity, or when a peroperative vena cava filter was used

Results of the sensitivity analysis excluding all variables related to thromboprophylaxis can be found in the Supplementary material 1. Compared with the primary analysis, the sensitivity analysis yielded effect estimates of similar magnitude and direction, with only minor differences between the two models.

## Discussion

This nationwide study is the first to assess the association between both patient-related factors and surgical and stapling techniques with postoperative hemorrhage rates in primary MBS. In total, there were 1.021 postoperative hemorrhages recorded (CD grade ≥ 2), yielding a postoperative hemorrhage rate of 1.2%. Surgical reintervention was performed for 67% of postoperative hemorrhages in patients after SG compared to 30.9% after bypass procedures. Multilevel analysis showed associations with postoperative hemorrhage in MBS for BMI, stroke or TIA in medical history, hypertension and liver disease.

The overall postoperative hemorrhage rate of 1.2% in this study is at the lower end of the range reported in international literature [6, 13]. This relatively low incidence likely reflects the standardized care and high-volume nature of MBS in the Netherlands, where the use of uniform protocols and specialized centers minimizes technical variation and enhances overall patient safety.

Regarding risk factors, our findings both align with and diverge from existing evidence. Consistent with previous studies [3, 13, 14], cardiovascular and systemic comorbidities, specifically hypertension, a history of stroke or TIA, and liver disease, were associated with postoperative hemorrhage. These conditions likely indicate underlying vascular fragility or impaired coagulation. It is highly probable that these high-risk groups also represent the subset of patients requiring anticoagulant therapy, which is associated with postoperative hemorrhage [5, 15, 16]. However, an important limitation of this study is that the DATO registry currently does not record specific data on the use of such medications, precluding a direct analysis of their impact on hemorrhage rates.

Notably, a lower BMI was associated with an increased risk of postoperative hemorrhage, a finding consistent with previous studies [3, 5]. This association may be explained by differences in tissue thickness and composition; patients with a lower BMI often have less visceral fat and thinner gastric walls. This can affect the interaction between surgical staples and tissue, potentially leading to inadequate vessel compression if the staple height is not calibrated.

In contrast to some large-scale datasets [7, 13, 16], factors such as age and sex were not associated with hemorrhage in our analysis. The discrepancy may be explained by the use of multilevel analysis, which allows for thorough adjustment of comorbidities. Since conditions like cardiovascular disease are often more prevalent in older and male populations, it is possible that these clinical factors, rather than age or sex alone, are the primary drivers of hemorrhage risk. Furthermore, SG has traditionally been reported to be associated with a higher incidence of postoperative hemorrhage than gastric bypass procedures. While the absolute hemorrhage rate for SG in our study was slightly higher, multilevel analysis showed that the procedure type itself was not an independent risk factor. This may suggest that the theoretically higher risk of the extensive SG staple line may be neutralized by surgical standardization in high-volume centers. The modest absolute difference suggests that, although a clinical trend exists, procedure type is not a significant independent driver of hemorrhage when adjusted for patient-specific factors.

Data for the additional surgical parameters in this study were collected via targeted questionnaires sent to all Dutch bariatric centers and subsequently linked to the DATO registry. This cross-sectional coupling enabled a comprehensive assessment of perioperative management, including thromboprophylaxis, TXA, and stapling materials. The questionnaires revealed considerable clinical heterogeneity across centers; for instance, there was substantial variation in the use of powered versus manual staplers (59.6% vs. 39.8%) and the duration of postoperative thromboprophylaxis, ranging from only during hospital admission to over four weeks.

In the primary analysis of our study, there was no difference in hemorrhage rates between the stapler brands. A significant difference has been found in a previous study [17]. In addition, we did not find a difference between the powered and manual stapling devices, in contrast to what has been reported previously [18]. Although the collected data of the brand and type of stapling device were validated by the various stapling device manufacturers, data regarding specific cartridges lacked the precision required for a detailed analysis. Even though we collected the type of cartridges that surgeons generally use in the questionnaire, the actual type of cartridges used is also dependent on the perioperative course. Therefore, the amount and type of cartridges used for each procedure could not be determined with certainty. However, we do recognize that this may play an important role in postoperative hemorrhages in primary MBS, as has been reported previously [19–22].

Sensitivity analysis was performed given the initial concerns about the reliability of the thromboprophylaxis questionnaire responses. As subsequent verification confirmed that no changes in thromboprophylaxis agent had occurred across the study period, this variable was therefore included in the final analysis. The sensitivity analysis showed partially different statistical significance patterns, however effect estimates remained largely consistent with the primary model, supporting the stability of the findings. Nevertheless, future research should focus on prospective data collection; for example, by integrating additional surgical variables, such as cartridges, staple height, compression duration, staple-line reinforcements, and information on use of anticoagulation and duration of thromboprophylaxis, into the national registry. Recording these detailed technical variables is essential to determine whether specific perioperative techniques or protocols can effectively mitigate hemorrhage risk.

While this study provides valuable insights based on real-world data and a large study population, certain limitations should be acknowledged. Although the core dataset was collected prospectively, the retrospective questionnaire could have introduced recall bias regarding the timeframe when specific surgical techniques, perioperative TXA protocols, and thromboprophylaxis regimens were implemented. Due to the registry-based design, with most data originating from the DATO registry, several variables of interest were not recorded. These include, for example, anticoagulation use in the presence of comorbidities and specific anaesthetic protocols, such as target procedural blood pressure and fluid management. In addition, the prevalence of certain comorbidities not associated with obesity appears lower than expected, which could be due to underreporting or missing values. Finally, there may be underreporting of CD grade 2 postoperative hemorrhages due to inter-center variation in the registration of such events and in the threshold for initiating pharmacological treatment, which itself defines this grade.

Nevertheless, with its large cohort and centralised, protocolised metabolic and bariatric care, this study provides relevant insights into how the use of different surgical techniques, type and brand of stapling devices, TXA protocols, and thromboprophylaxis regimens are associated with postoperative hemorrhage in primary MBS. The factors BMI, stroke or TIA in medical history, hypertension, and liver disease were significantly associated with postoperative hemorrhage. There was no association between stapler material or surgical technique and postoperative hemorrhages. Even though the risk of postoperative hemorrhages are low in primary MBS, this study aids in identifying patients with an increased risk of postoperative hemorrhage, enabling preventive measures to reduce the risk of postoperative hemorrhage even further.

## Supplementary Information

Below is the link to the electronic supplementary material.

Supplementary file1 (DOCX 35 KB)

## Data Availability

The data that support the findings of this study are available from the corresponding author upon reasonable request.
