# Supplementary material for: Factors Associated with Postoperative Hemorrhage in Primary Metabolic Bariatric Surgery: Nationwide Analysis of Patient Characteristics, Surgical and Stapling Techniques
Source: Obes Surg. 2026 Oct 2;36(11):5752–61. doi: 10.1007/s11695-026-08930-9 (PMC13714033; doi:10.1007/s11695-026-08930-9)
Supplement: Supplementary file 1 [file 11695_2026_8930_MOESM1_ESM.docx]

| **Predictors** |  | **Crude odds ratio** | | **95% CI OR** | **p-value** | **Adjusted odds ratio** | **95% CI OR** | **p-value** |
| --- | --- | --- | --- | --- | --- | --- | --- | --- |
| **Sex** | Male (ref) | Ref. |  | |  | Ref. |  |  |
|  | Female | 0.633 | | 0.554 - 0.726 | <0.001 | 0.805 | 0.657 - 0.986 | 0.036 |
| **Age (per year)** |  | 1.024 | | 1.018- 1.029 | <0.001 | 1.098 | 1.088 - 1.108 | 0.036 |
| **BMI (kg/m2) (for each BMI point)** |  | 0.965 | | 0.953-0.978 | <0.001 | 1.175 | 1.157 - 1.193 | 0.003 |
| **Type of surgery** | Sleeve gastectomy | Ref. | |  |  | Ref. |  |  |
|  | Bypass variation* | 0.647 | | 0.567-0.741 | <0.001 | 0.695 | 0.561 - 0.861 | <0.001 |
| **ASA score** | 1 | Ref. | |  |  |  |  |  |
|  | 2 | 1.443 | | 0.736-3.388 | 0.339 |  |  |  |
|  | 3 | 1.643 | | 0.842-3.843 | 0.193 |  |  |  |
|  | 4 | 1.862 | | 0.690-5.234 | 0.220 |  |  |  |
| **Increased risk VTE/PE** | No increased risk | Ref. | |  |  |  |  |  |
|  | Increased risk | 1.158 | | 0.410-2.538 | 0.748 |  |  |  |
| **Stroke/TIA in medical history** | No | Ref. | |  |  | Ref. |  |  |
|  | Yes | 1.973 | | 1.313-2.842 | <0.001 | 1.559 | 1.007 - 2.413 | 0.047 |
| **Peripheral arterial disease / AAA** | No | Ref. | |  |  | Ref. |  |  |
|  | Yes | 2.639 | | 1.468-4.350 | <0.001 | 1.483 | 0.796 - 2.763 | 0.215 |
| **Diabetes** | No | Ref. | |  |  | Ref. |  |  |
|  | Yes | 1.338 | | 1.149-1.551 | <0.001 | 1.090 | 0.865 - 1.372 | 0.465 |
| **Hypertension** | No | Ref. | |  |  | Ref. |  |  |
|  | Yes | 1.605 | | 1.417-1.818 | <0.001 | 1.417 | 1.161 - 1.729 | <0.001 |
| **Kidney disease** | No | Ref. | |  |  |  |  |  |
|  | Yes | 1.307 | | 0.465-2.850 | 0.555 |  |  |  |
| **Myocardial infarction in medical history** | No | Ref. | |  |  | Ref. |  |  |
|  | Yes | 2.192 | | 1.531-3.039 | <0.001 | 1.307 | 0.837 - 2.040 | 0.239 |
| **Liver disease** | No | Ref. | |  |  | Ref. |  |  |
|  | Yes | 2.157 | | 1.201-3.550 | 0.005 | 1.830 | 1.054 - 3.175 | 0.032 |
| **Malignancy in medical history** | No | Ref. | |  |  |  |  |  |
|  | Yes | 1.333 | | 0.686-2.314 | 0.349 |  |  |  |
| **OSAS** | No | Ref. | |  |  | Ref. |  |  |
|  | Yes | 1.407 | | 1.214-1.625 | <0.001 | 1.158 | 0.934 - 1.435 | 0.181 |
| **Dyslipidaemia** | No | Ref. | |  |  | Ref. |  |  |
|  | Yes | 1.373 | | 1.188-1.583 | <0.001 | 0.955 | 0.761 - 1.198 | 0.690 |
| **Congestive heart failure** | No | Ref. | |  |  | Ref. |  |  |
|  | Yes | 1.724 | | 1.148-2.481 | 0.005 | 1.070 | 0.673 - 1.701 | 0.776 |
| **Preoperative thromboprophylaxis** | No | Ref. | |  |  |  |  |  |
|  | Yes | 0.733 | | 0.619-0.862 | <0.001 |  |  |  |
| **Tromboprophylaxis agent** | Dalteparin | Ref. | |  |  |  |  |  |
|  | Nadroparin | 0.882 | | 0.772-1.007 | 0.064 |  |  |  |
| **Duration thromboprophylaxis** | Only during hospital admission | Ref. | |  |  |  |  |  |
|  | 1 week | 0.487 | | 0.372-0.628 | <0.001 |  |  |  |
|  | 2 weeks | 0.631 | | 0.494-0.797 | <0.001 |  |  |  |
|  | 3 weeks | 1.140 | | 0.896-1.435 | 0.275 |  |  |  |
|  | 4 or more weeks | 0.689 | | 0.598-0.795 | <0.001 |  |  |  |
| **Dosage thromboprophylaxis** | Single dose | Ref . | |  |  |  |  |  |
|  | Double dose | 1.510 | | 0.484-9.123 | 0.562 |  |  |  |
|  | Therapeutic dose | 4.796 | | 0.219-51.671 | 0.207 |  |  |  |
|  | 7500EH dalteparin | 2.318 | | 0.731-14.089 | 0.240 |  |  |  |
| **Standardized TXA pre- or peroperatively** | No standard use | Ref. | |  |  |  |  |  |
|  | Standard use | 1.039 | | 0.411-2.126 | 0.925 |  |  |  |
| **Omentopexy in Sleeve Gastrectomy** | No | Ref. | |  |  |  |  |  |
|  | Yes | 0.905 | | 0.323-1.963 | 0.824 |  |  |  |
| **Brand stapling device** | A | Ref. | |  |  | Ref. |  |  |
|  | B | 0.880 | | 0.763-1.011 | 0.075 | 0.610 | 0.431 - 0.863 | 0.005 |
|  | C | 0.780 | | 0.494-1.164 | 0.254 | 1.003 | 0.581 - 1.729 | 0.993 |
| **Type of stapling device** | Powered | Ref. | |  |  | Ref. |  |  |
|  | Manual | 0.873 | | 0.767-0.991 | 0.037 | 0.836 | 0.623 - 1.123 | 0.235 |
| **Year** | 2015 | Ref. | |  |  | Ref. |  |  |
|  | 2016 | 0.994 | | 0.737-1.345 | 0.971 | 2.567 | 0.270 - 24.379 | 0.412 |
|  | 2017 | 1.041 | | 0.776-1.400 | 0.791 | 1.039 | 0.090 - 12.018 | 0.975 |
|  | 2018 | 1.136 | | 0.849-1.526 | 0.393 | 0.601 | 0.078 - 4.618 | 0.624 |
|  | 2019 | 0.985 | | 0.735-1.324 | 0.918 | 0.444 | 0.059 - 3.323 | 0.429 |
|  | 2020 | 1.109 | | 0.809-1.520 | 0.519 | 0.516 | 0.069 - 3.873 | 0.520 |
|  | 2021 | 1.146 | | 0.852-1.547 | 0.369 | 0.476 | 0.063 - 3.579 | 0.471 |
|  | 2022 | 1.192 | | 0.902-1.584 | 0.221 | 0.497 | 0.066 - 3.729 | 0.050 |
|  | 2023 | 1.329 | | 1.003-1.770 | 0.050 | 0.589 | 0.079 - 4.414 | 0.607 |
|  | 2024 | 1.340 | | 1.007-1.793 | 0.046 | 0.621 | 0.083 - 4.660 | 0.643 |

**Supplement 1. Sensitivity analysis**

Univariable analyses present the unadjusted association between each predictor and the post-operative hemorrhage rate (CD ≥ 2). For this sensitivity analysis, all variables related to thromboprophylaxis, including the specific agent use, were excluded from the multilevel model to account for potential limitations in data quality.

Abbreviations: CI = confidence interval; BMI = body mass index; kg/m^2^ = kilogram per squared meters; OSAS = obstructive sleep apnoea syndrome; ASA= American Society of Anesthesiologists; AAA = abdominal aortic aneurysm ; TIA = transient ischemic attack; VTE = venous thromboembolism; PE = pulmonary embolism; TXA = tranexamic acid. *Bypass variations: Roux-en-Y gastric bypass, one anastomosis gastric bypass and ring-augmented RYGB and OAGB. ** Increased risk of VTE/PE was defined as a venous thromboembolism or pulmonary hypertension in medical history, hypoventilation due to obesity, or when a perioperative vena cava filter was used.
